# Fatal Case of Intus-Susception in a Child

**Published:** 1828-06

**Authors:** Edward Humpage


INTROSUSCEPTION.

*Fatal Case of Intus-susception in a Child.* By Edward Humpage, Esq.

I was summoned, on the 6th March, to visit a child, about two years and a half old, reported to be in a fit. On arriving, I found the little patient just taken from a warm bath, and recovering from what appeared a spasmodic affection more resembling tetanus than any thing else. I administered some warm water, and, finding that the child had eaten a hearty dinner, prescribed an emetic mixture, containing Ipecac. comp. ℈j. with Ant. Tart. gr. ij., to be given in divided doses.

I was again called in half an hour, another spasm having come on. The family physician had now arrived. We immersed the child in warm water, and agreed to open the jugular vein, the emetic not having produced any effect. After about six ounces of blood had flowed, the rigidity ceased; then followed occasional eructations, but no vomiting or evacuation by the bowels. The child lay quietly, drank frequently of warm water, and seemed in all respects relieved. This state of things was, however, but of short duration; for in about three hours another spasm came on, which the bath and two turpentine clysters in some degree relieved, (the child having after them several stools;) but constant subsultus tendinum followed, and death closed the scene in about seven hours from the first attack.

On examining the body, we found the lining membrane of the abdomen, and all the viscera, perfectly healthy, excepting at the commencement of the ileum, to which spot our attention was directed by the increased vascularity of its mesenteric blood-vessels. On more minute inspection, we found there was an introsusception downwards, of about one inch and a half in extent. I easily withdrew the included portion of gut, which (if I may use the expression) was not strangulated, but incarcerated; its villous and peritoneal coats were highly injected with red vessels, the upper portion being much contracted, the lower somewhat more than its usual size.

That this child died from the invagination of intestine, I think is very evident; and the urgent symptoms it produced teach us the remarkable sympathy of the ganglionic with the spino-cerebral system of nerves, the irritation commencing in the former, and extending itself through the whole locomotive system. The organs of deglutition were but partially affected: nor is this circumstance surprising, when we remember the superadded nerves which supply this part. The absence of vomiting is not easily accounted for, cases of this sort being generally accompanied by this symptom. On inquiry, I find this child had, some few months ago, an attack of colic, with vomiting and purging, which probably was the effect of invagination to a slight degree; the intestine resuming its natural position by the increased action of the stomach and bowels, leaving a predisposition to a recurrence of the disease, which probably was favored by the quantity and quality of the food taken on the day of the fatal attack.

*Bristol; March 10th,* 1828.

---

